# Ulcerative colitis-associated bronchiectasis: A rare extraintestinal manifestation of inflammatory bowel disease: A case report

**DOI:** 10.1097/MD.0000000000030203

**Published:** 2022-08-26

**Authors:** Marouf Alhalabi, Sawsan Ali Deeb, Fadwa Ali, Ahmad Abbas

**Affiliations:** a Gastroenterology department of Damascus hospital, Damascus, Syria.

**Keywords:** a case report, bronchiectasis, extraintestinal manifestations, inflammatory bowel disease, ulcerative colitis

## Abstract

Inflammatory bowel disease patients may suffer from extraintestinal manifestations. Although muscles, joints, and skin are the most commonly affected, respiratory involvement is more prevalent than previously believed, and the majority of these patients have no symptoms. Although the large airways are the most frequently affected, the small airways, lung parenchyma, and pulmonary vasculature may also be affected.

**Patient concerns:**

A 24-year-old nonsmoking Syrian female was referred to the pulmonary medicine clinic in December 2020 due to a chronic cough. Her cough had been present for the last year, it was described as scratchy, and produced small amounts of mucoid sputum occasionally. She denied any related wheeze, hemoptysis, weight loss, or night sweats. Multiple courses of antibiotics were prescribed by many doctors, also previous chest radiographs were reported as normal. She was diagnosed with ulcerative colitis in 2012 after presentation with abdominal pain and per rectal bleeding. The diagnosis was confirmed via colonoscopy and colon biopsies, with no prior surgery. Her past medications included prednisone, mesalamine, azathioprine, and infliximab. Tests, including complete blood count, C-reactive protein (CRP), fecal calprotectin, and chest X-ray, were normal.

**Diagnosis:**

Ulcerative colitis-associated bronchiectasis was established through history and clinical examination beside pulmonary function test, which revealed a mild obstructive pattern, and a chest computed tomography follow-up that revealed bilateral bronchiectasis.

**Interventions:**

Bronchiectasis was treated with inhaled oral steroids and sputum expectoration while she continued mesalamine and azathioprine for ulcerative colitis.

**Outcome:**

Cough improvement and sustained ulcerative colitis remission.

**Conclusions:**

Identification of inflammatory bowel disease pulmonary exacerbation is probably poor, as pulmonary symptoms might emerge at any moment during the illness, and are most commonly diagnosed later in life and with the disassociation of inflammatory bowel disease activity. Pulmonologists should be involved in the care of inflammatory bowel disease patients who developed lung symptoms.

## 1. Introduction

extraintestinal manifestations of inflammatory bowel disease are common and can occur before or after the disease has been diagnosed. They commonly affect the joints, skin, and eyes, but they can also affect other organs such as the liver, lungs, and pancreas.^[1]^ Certain extraintestinal manifestations, such as oral aphthous ulcers, peripheral arthritis, erythema nodosum, or episcleritis are often related to active intestinal inflammation and typically improve when the intestinal disease is treated.^[1]^ Other extraintestinal manifestations, such as uveitis or ankylosing spondylitis, are usually unrelated to gastrointestinal inflammatory activity.^[1]^ Less common, extraintestinal manifestations, such as pyoderma gangrenosum and primary sclerosing cholangitis, have an ambiguous relationship with underlying inflammatory bowel activity.^[2]^ It is approximated that 40% to 60% of IBD patients have abnormal pulmonary function tests, but the majority of these patients are asymptomatic.^[3]^ The identification of IBD’s respiratory manifestations is poor, and the incidence is underestimated.^[4]^ They may impact the large and small airways, lung parenchyma, and pulmonary vasculature.^[3]^ Chronic bronchitis, chronic bronchial suppuration, and bronchiectasis are the most common large airway diseases in UC,^[5]^ and appear years after UC onset,^[6]^ and seldom precede UC diagnosis.^[6]^ The diagnostic difficulties of IBD pulmonary manifestations are exacerbated by presentation heterogeneity, variability in the time frame of respiratory symptoms regarding IBD onset, and low prevalence of clinical signs of airway involvement.^[7]^ Understanding and early recognition of this relationship may help gastroenterologists, pulmonologists, and general practitioners to treat and prevent the development of respiratory disease. Bronchiectasis may be the most serious clinically, as irreversible structural changes may place patients at risk for severe frequent respiratory tract infections, also IBD-associated bronchiectasis responds well to inhaled corticosteroid.^[8]^

## 2. Case presentation

**Figure 1.**
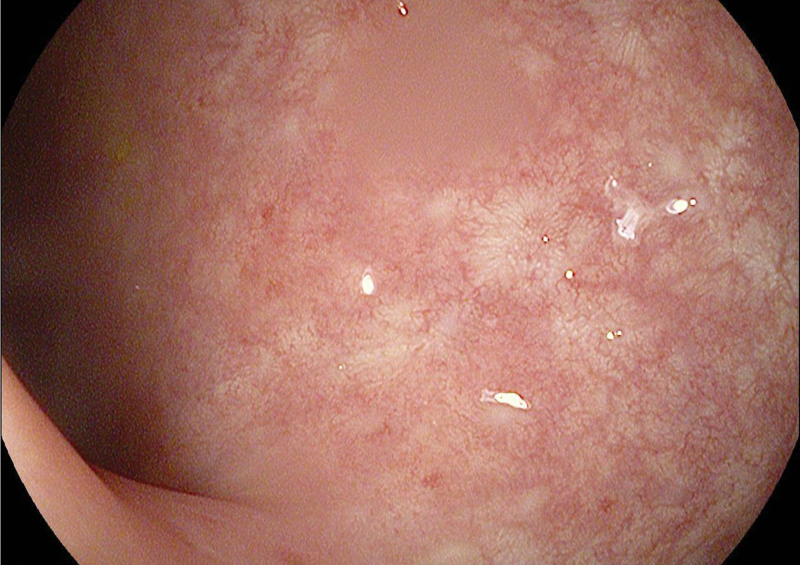
Colonoscopy revealed erythema and a decreased vascular pattern.

**Figure 2.**
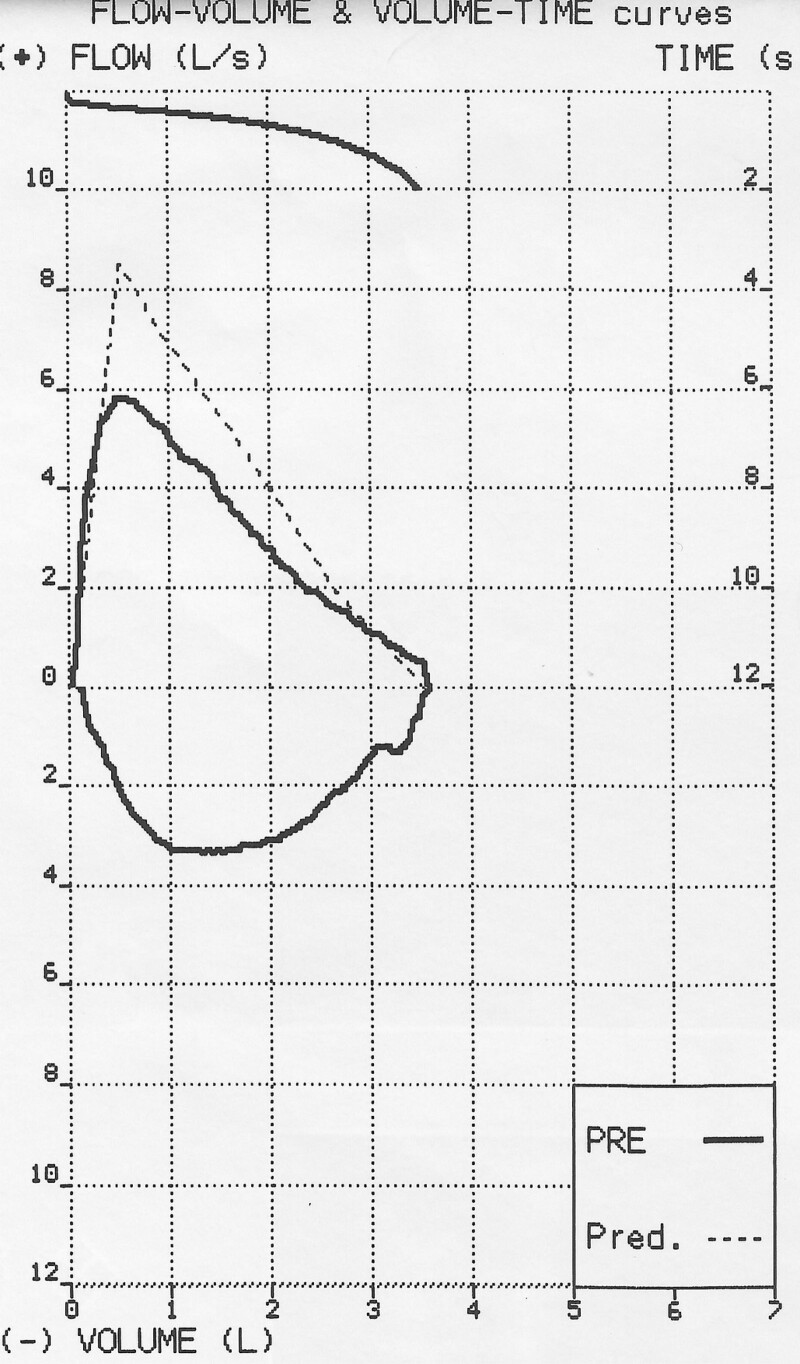
Pulmonary function testing demonstrating a mild obstructive pattern.

**Figure 3.**
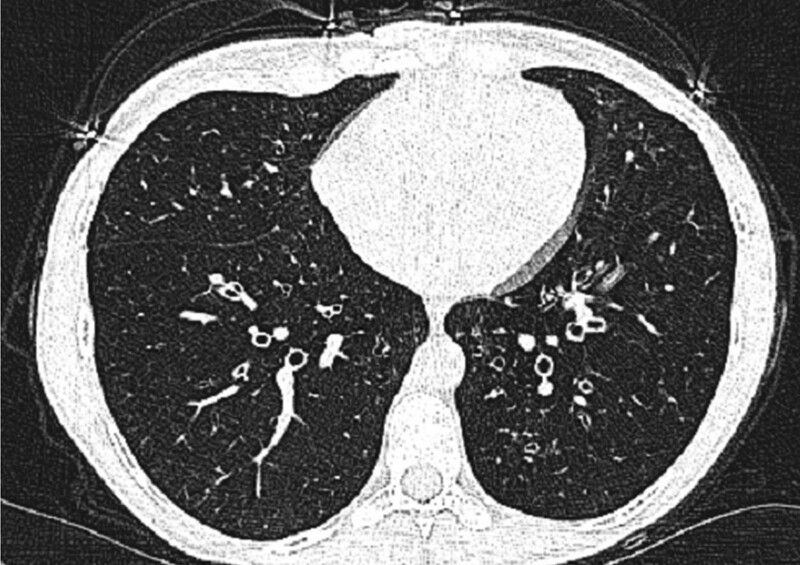
Chest computed tomography revealed bilateral bronchiectasis.

A 24-year-old Syrian female was referred to the pulmonary clinic in December 2020 due to a chronic cough. Her cough had been presented for past year and produced small amounts of mucoid sputum occasionally. She denied any related wheeze, hemoptysis, weight loss, or night sweats. Multiple courses of antibiotics were prescribed by many doctors, also previous chest radiographs were reported as normal. She was a nonsmoker for her entire life, with no known exposure to passive smoking, domestic animals, or potential occupational precipitants. There was no family history of respiratory disease. She was diagnosed with ulcerative colitis in 2012 after presentation with abdominal pain and rectal bleeding. Colonoscopy and colon biopsies confirmed the diagnosis, she had no prior surgery. Her past medications included prednisone 40 mg during flares then tapering; mesalamine up to 3 g/day; azathioprine up to 2 mg/kg per day; and infliximab at a dose of 5 mg/kg at 0, 2, and 6 weeks, followed by 5 mg/kg every 8 weeks for steroid-dependent ulcerative colitis.^[9]^ She has had 13 doses, including induction from April 2012 until July 2014 then infliximab stopped due to unavailability. Her routine surveillance colonoscopies (the newest one was about ten months ago), revealed erythema and a decreased vascular pattern figure (1),^[10–14]^ with the initial colonoscopy revealing pancolitis. She had normal nonbloody stool frequency, she scored 1 point on the Mayo score.^[15]^ When we are currently evaluating her, she had not any complaints, she scored zero points on the partial Mayo scale (which means that she was in remission).^[15]^ Tests, including complete blood count, C-reactive protein (CRP), fecal calprotectin, purified protein derivative skin, immunoglobulin IgA, IgG, IgM, and IgE were all within normal limits^[16]^; also chest X-ray was reported as normal. Pulmonary function testing demonstrated a mild obstructive pattern table (1) and figure (2) because she responded to first-line treatment in the outpatient clinic, no sputum samples were collected for microscopy, culture and sensitivities, or cell differential analysis. Furthermore, no bronchoscopy or lung biopsy was performed. A high-resolution CT scan of the chest revealed cylindrical bronchiectasis in segmental and subsegmental bronchi, figure (3). She continued with mesalamine and azathioprine and began the administration of inhaled corticosteroid therapy.^[1]^ Three months after the initial diagnosis, she had a good clinical response with cough remission and without sputum production. Inhaled corticosteroid treatment was sustained for another 6 months after her improvement, then she began to taper inhaled corticosteroid. This did not result in a recurrence of her symptoms, and her inhaled steroid therapy was stopped with intermittent use when the cough exacerbates.

## 3. Discussion

IBD is linked to several extraintestinal comorbidities, in UC, around 31% of patients had at least 1 extraintestinal manifestation.^[17]^ Pulmonary manifestations may affect large airways which are common, although still rare.^[18]^ While bronchopulmonary involvements express themselves in several ways with bronchial tree airway inflammation is the most prevalent pattern, and bronchiectasis is the most commonly reported disorder.^[19]^ More notably, there are a variety of subclinical respiratory irregularities that are frequently overlooked in regular medical assessments. Furthermore, pulmonary embolism and deep venous thrombosis can emerge because of inflammatory bowel disease.^[20]^ The pathophysiological pathway underlying ulcerative colitis-associated bronchiectasis is still unknown. Histopathological findings in respiratory tissues appear to reflect those in the digestive tract, including epithelial changes, neutrophilic infiltrates, and subepithelial gland damage.^[21]^ There are many causes of chronic cough, gastroesophageal reflux disease,^[22–25]^ postnasal drip,^[24,25]^ and cough-variant asthma,^[24,25]^ which were ruled out as there were no related clinical signs. Where it is known that UC treatments such as corticosteroids, azathioprine, and antitumor necrosis factor-alpha (antiTNFα) may predispose to the development of opportunistic infections.^[26]^ Acute respiratory infection was ruled out because her CBC/WBC, CRP, and chest X-ray were all normal. A chronic lung infection such as pulmonary tuberculosis or chronic aspergillosis was also ruled out as there were no systemic symptoms and tests and chest X-rays were all normal. Chronic bacterial bronchitis was unlikely because of response failure to many antibiotics courses. UC medication could use many pulmonary diseases, 5-ASA medicines produce interstitial lung disease,^[19,27–29]^ and mesalamine-induced lung toxicity.^[27–29]^ Mesalamine is less pneumotoxic than other IBD medications, such as methotrexate and sulfasalazine, but the lack of eosinophilia and improvement in symptoms despite continued mesalamine treatment rule out mesalamine-induced toxicity/mesalazine-induced bronchiectasis. Methotrexate could cause pulmonary fibrosis, hypersensitivity pneumonitis, pulmonary eosinophilia, and organizing pneumonia,^[30–35]^ but she was not receiving methotrexate. antiTNFα produces granulomatous inflammation,^[36]^ but she had stopped infliximab several years ago. Many rare diseases may affect the central airways, such as Wegener granulomatosis, microscopic polyangiitis,^[18]^ and amyloidosis,^[37]^ however, it should result in relating abnormalities in lung function or radiology, lung function test, and CT scan. Finally, colectomy may be a risk factor for pulmonary involvement,^[7]^ but she had no prior surgery at all. As the CT scan revealed bronchiectasis, there is no clinical or biochemical evidence for a cause of bronchiectasis other than UC implying that her clinical condition was secondary to its presence.^[18]^ Treatment aims to alleviate symptoms such as coughing, sputum production, and dyspnea while also preventing further airway damage.^[38]^ The treatment’s success is dependent on treating an identifiable underlying condition, as well as drugs to enhance bronchodilation and mucociliary clearance, such as secretory mobilization techniques (chest physiotherapy) and mucolytic agents, recurrent infections can be treated with medication, or in rare cases, surgery can be utilized to treat localized disease.^[39]^ According to a European Crohn and Colitis guideline, inhaled corticosteroids should be used as first-line in large airways disease, whereas oral therapy is held for parenchymal involvement or inhaled steroid-resistant large airways disease. While immunomodulating or/and biological therapy are preserved for refractory disease. Treatment success is linked to improved symptoms, pulmonary function, and cellular decrease in neutrophilia. Although imaging may reveal improvements in the level of air trapping.^[40]^

This case exemplifies key aspects of the diagnostic challenge in such patients, particularly the gradual onset of symptoms and the dissociation of the first presentation of colonic and airway disease symptoms. It also shows that inhaled corticosteroid was effective in patients with early bronchiectasis. Pulmonologists should be involved in the care of inflammatory bowel disease patients who developed lung symptoms as pulmonary symptoms might emerge at any moment during the illness.

**Table 1:** Lung function results from the patient prior to treatment.

Spirometry	Measured	% Predicted
PEF L/s	5.85	68
FEV1 (L)	2.98	96
FIVC (L)	3.40	95
FEV1/FVC	84.2	97
FEF 25%	5.22	72
FEF50%	3.13	66
FEF 75%	1.58	79

FEF = forced expiratory flow, FEV1 = forced expiratory volume, FIVC = forced inspiratory vital capacity, PEF = peak expiratory flow.

## Author contributions

All authors participated in the management of the patient described in this case report. Dr.Marouf Alhalabi collected all the references and was a major contributor in the writing of the article. All authors have read and approved the article.

Conceptualization: Dr.Marouf Alhalabi

Investigation: Dr.Marouf Alhalabi

Resources: Dr.Marouf Alhalabi

Supervision: Dr.Marouf Alhalabi

Validation: Dr.Marouf Alhalabi

Writingoriginal draft: Dr.Marouf Alhalabi

Writingreview and editing: Marouf Mouhammad Alhalabi, Sawsan Ali Deeb, Fadwa Ali, Ahmad Abbas

## Acknowledgments

All authors have read and approved the manuscript, on behalf of all the contributors I will act as guarantor and will correspond with the journal from this point onward.

## References

[1] Harbord M Annese V Vavricka SR. The first european evidence-based consensus on extra-intestinal manifestations in inflammatory bowel disease. J Crohn’s Colitis. 2016;10:239–54. 26614685 10.1093/ecco-jcc/jjv213 PMC4957476

[2] Vavricka SR Schoepfer A Scharl M. Extraintestinal manifestations of inflammatory bowel disease. Inflamm Bowel Dis. 2015;21:1982–92. 26154136 10.1097/MIB.0000000000000392 PMC4511685

[3] Cozzi D Moroni C Addeo G. Radiological patterns of lung involvement in inflammatory bowel disease. Gastroenterol Res Pract. 2018;2018:1e5697846–10. 10.1155/2018/5697846 PMC6109524 30158965

[4] Papanikolaou I Kagouridis K Papiris SA. Patterns of airway involvement in inflammatory bowel diseases. World J Gastrointest Pathophysiol. 2014;5:560–9. 25400999 10.4291/wjgp.v5.i4.560 PMC4231520

[5] Black H Mendoza M Murin S. Thoracic manifestations of inflammatory bowel disease. CHEST. 2007;131:524–32. 17296657 10.1378/chest.06-1074

[6] Mahadeva R Walsh G Flower CD. Clinical and radiological characteristics of lung disease in inflammatory bowel disease. Eur Respir J. 2000;15:41–8. 10678619 10.1183/09031936.00.15104100

[7] Camus P Colby TV. The lung in inflammatory bowel disease. Eur Respir J. 2000;15:5–10. 10678613 10.1183/09031936.00.15100500

[8] D’Andrea N Vigliarolo R Sanguinetti CM. Respiratory involvement in inflammatory bowel diseases. Multidiscip Respir Med. 2010;5:173–82. 22958334 10.1186/2049-6958-5-3-173 PMC3463044

[9] Harbord M Eliakim R Bettenworth D. Third european evidence-based consensus on diagnosis and management of ulcerative colitis. Part 2: current management. J Crohn’s Colitis. 2017;11:769–84. 28513805 10.1093/ecco-jcc/jjx009

[10] Clarke WT Feuerstein JD. Colorectal cancer surveillance in inflammatory bowel disease: practice guidelines and recent developments. World J Gastroenterol. 2019;25:4148–4157. 31435169 10.3748/wjg.v25.i30.4148 PMC6700690

[11] Cairns SR Scholefield JH Steele RJ. Guidelines for colorectal cancer screening and surveillance in moderate and high risk groups (update from 2002). Gut. 2010;59:666–89. 20427401 10.1136/gut.2009.179804

[12] Magro F Gionchetti P Eliakim R. Third european evidence-based consensus on diagnosis and management of ulcerative colitis. Part 1: definitions, diagnosis, extra-intestinal manifestations, pregnancy, cancer surveillance, surgery, and ileo-anal pouch disorders. J Crohns Colitis. 2017;11:649–70. 28158501 10.1093/ecco-jcc/jjx008

[13] Basseri RJ Basseri B Vassilaki ME. Colorectal cancer screening and surveillance in Crohn’s colitis. J Crohn’s Colitis. 2012;6:824–9. 22398087 10.1016/j.crohns.2012.01.005

[14] Farraye FA Odze RD Eaden J. AGA technical review on the diagnosis and management of colorectal neoplasia in inflammatory bowel disease. Gastroenterology. 2010;138:746–74, 774.e1. 20141809 10.1053/j.gastro.2009.12.035

[15] Sturm A Maaser C Calabrese E. ECCO-ESGAR guideline for diagnostic assessment in IBD part 2: IBD scores and general principles and technical aspects. J Crohn’s Colitis. 2019;13:273–84. 30137278 10.1093/ecco-jcc/jjy114

[16] Schussler E Beasley MB Maglione PJ. Lung disease in primary antibody deficiencies. J Allergy Clin Immunol Pract. 2016;4:1039–52. 27836055 10.1016/j.jaip.2016.08.005 PMC5129846

[17] Vavricka SR Brun L Ballabeni P. Frequency and risk factors for extraintestinal manifestations in the Swiss inflammatory bowel disease cohort. Am J Gastroenterol. 2011;106. 10.1038/ajg.2010.343 20808297

[18] Black H Mendoza M Murin S. Thoracic manifestations of inflammatory bowel disease. CHEST. 2007;131:524–32. 17296657 10.1378/chest.06-1074

[19] Storch I Sachar D Katz S. Pulmonary manifestations of inflammatory bowel disease. Inflamm Bowel Dis. 2003;9:104–15. 12769444 10.1097/00054725-200303000-00004

[20] Fumery M Xiaocang C Dauchet L. Thromboembolic events and cardiovascular mortality in inflammatory bowel diseases: a meta-analysis of observational studies☆. J Crohn’s Colitis. 2014;8:469–79. 24183231 10.1016/j.crohns.2013.09.021

[21] Mateer SW Maltby S Marks E. Potential mechanisms regulating pulmonary pathology in inflammatory bowel disease. J Leukoc Biol. 2015;98:727–37. 26307547 10.1189/jlb.3RU1114-563R

[22] Francis DO. Chronic cough and gastroesophageal reflux disease. Gastroenterol Hepatol (N Y). 2016;12:64–6. 27330507 PMC4865789

[23] Fontana GA Pistolesi M. Cough · 3: chronic cough and gastro-oesophageal reflux. Thorax. 2003;58:1092–5. 14645983 10.1136/thorax.58.12.1092 PMC1746530

[24] Morice AH Millqvist E Bieksiene K. ERS guidelines on the diagnosis and treatment of chronic cough in adults and children. Eur Respir J. 2019;55:1901136. 10.1183/13993003.01136-2019 PMC6942543 31515408

[25] Sylvester DC Karkos PD Vaughan C. Chronic cough, reflux, postnasal drip syndrome, and the otolaryngologist. Int J Otolaryngol. 2012;2012:564852. 22577385 10.1155/2012/564852 PMC3332192

[26] Rahier JF Magro F Abreu C. Second european evidence-based consensus on the prevention, diagnosis and management of opportunistic infections in inflammatory bowel disease. J Crohns Colitis. 2014;8:443–68. 24613021 10.1016/j.crohns.2013.12.013

[27] Price LC Poullis A Grubnic S. Mesalazine-induced bronchiectasis and eosinophilia in a patient with ulcerative colitis: a case report. J R Soc Med. 2007;100:151–2. 17339311 10.1258/jrsm.100.3.151 PMC1809164

[28] Bitton A Peppercorn MA Hanrahan JP. Mesalamine-induced lung toxicity. Am J Gastroenterol. 1996;91:1039–40. 8633548

[29] Kotsiou OS Gourgoulianis KI. A case report of mesalazine-induced lung injury: a reversible drug side effect. Respir Med Case Rep. 2019;27:100865. 10.1016/j.rmcr.2019.100865 PMC6545345 31193970

[30] Imokawa S Colby TV Leslie KO. Methotrexate pneumonitis: review of the literature and histopathological findings in nine patients. Eur Respir J. 2000;15:373–81. 10706507 10.1034/j.1399-3003.2000.15b25.x

[31] Conway R Low C Coughlan RJ. Methotrexate use and risk of lung disease in psoriasis, psoriatic arthritis, and inflammatory bowel disease: systematic literature review and meta-analysis of randomised controlled trials. BMJ. 2015;350:h1269. 25770113 10.1136/bmj.h1269 PMC4358852

[32] Solomon D Glynn R Karlson E. Adverse effects of low-dose methotrexate: a randomized trial. Ann Intern Med. 2020;172:369–80. 32066146 10.7326/M19-3369 PMC7229518

[33] Jakubovic BD Donovan A Webster PM. Methotrexate-induced pulmonary toxicity. Can Respir J. 2013;20:153–5. 23762881 10.1155/2013/527912 PMC3814259

[34] Howard SC McCormick J Pui CH. Preventing and managing toxicities of high-dose methotrexate. Oncologist. 2016;21:1471–82. 27496039 10.1634/theoncologist.2015-0164 PMC5153332

[35] Kremer JM Alarcón GS Weinblatt ME. Clinical, laboratory, radiographic, and histopathologic features of methotrexate-associated lung injury in patients with rheumatoid arthritis: a multicenter study with literature review. Arthritis Rheum. 1997;40:1829–37. 9336418 10.1002/art.1780401016

[36] Fiorino G Danese S Pariente B. Paradoxical immune-mediated inflammation in inflammatory bowel disease patients receiving anti-TNF-α agents. Autoimmun Rev. 2014;13:15–9. 23777821 10.1016/j.autrev.2013.06.005

[37] Tw B Cw E. Pulmonary nodules due to reactive systemic amyloidosis (AA) in Crohn’s disease. Thorax. 1993;48:1287–8. 8303644 10.1136/thx.48.12.1287 PMC465004

[38] Rosen MJ. Chronic cough due to bronchiectasis: ACCP evidence-based clinical practice guidelines. CHEST. 2006;129:122S–31S. 16428701 10.1378/chest.129.1_suppl.122S

[39] Hill AT Sullivan AL Chalmers JD. British thoracic society guideline for bronchiectasis in adults. Thorax. 2019;74(suppl 1):1–69. 10.1136/thoraxjnl-2018-212463 30545985

[40] Hamada S Ito Y Imai S. Effect of inhaled corticosteroid therapy on CT scan-estimated airway dimensions in a patient with chronic bronchitis related to ulcerative colitis. CHEST. 2011;139:930–2. 21467060 10.1378/chest.10-1105

